# Housing tenure and hospital admissions for acute lower respiratory tract infections in children less than 2 years: A Scottish birth cohort (2010-2012)

**DOI:** 10.23889/ijpds.v8i2.2232

**Published:** 2023-09-14

**Authors:** Caroline Hart, Samantha Hajna, Bianca De Stavola, Tom Clemens, Christopher Dibben, Steven Cunningham, Alison Macfariane, Jonathon Taylor, Linda Wijlaars, Pia Hardelid

**Affiliations:** 1 University College London, Great Ormond Street Institute of Child Health, London, United Kingdom; 2 Brock University, Department of Health Sciences, St. Catharines, Canada; 3 The University of Edinburgh, School of Geosciences, Edinburgh, United Kingdom; 4 The University of Edinburgh, MRC centre for Inflammation Research, Edinburgh, United Kingdom; 5 City, University of London, School of Health Sciences, London, United Kingdom; 6 Tampere University, Department of Civil Engineering, Tampere, Finland

## Objectives

To estimate the association between household tenure and the odds of hospital admission for acute lower respiratory tract infections (LRTI) in children under age 2 years.

## Method

We developed a birth cohort of all singleton children born in Scotland 2010-2012, using linked birth registration records and maternal Census 2011 data. Further linkage to hospital admission records provided information on acute LRTI (pneumonia, bronchitis, bronchiolitis, influenza, unspecified LRTI) admissions in children aged less than 2 years. Using logistic regression models, we estimated the association between housing tenure at birth (owned, social rented, private rented/lives rent free) with odds of hospital admission for LRTI before and after adjustment for parental occupational class (household reference), family type and highest qualification level.

## Results

From the cohort of all 174,279 births in 2010-2012, 84.1% linked to a maternal census record. Children whose parents were married or had a UK-born mother were more likely to link to a Census record. In the final linked cohort of 141,336 children, 7,486 (5.3%) were admitted to hospital for one or more LRTI during the 2 years of follow up. We found an association between housing tenure and LRTI admissions, with children residing in social rented, compared to owned housing having higher odds of an LRTI admission, OR: 1.40 (1.32-1.47); and children living in private rented/rent free housing, compared to owned, OR: 1.18 (1.11-1.26). After adjustment for household socioeconomic circumstances, these estimates attenuated to OR: 1.18 (1.11-1.27) and OR: 1.10 (1.03-1.18) respectively.

## Conclusion

After accounting for household socioeconomic circumstances, children living in social and private tenured accommodation, compared to children living in owned accommodation were more likely to be hospitalised for an acute LRTI during the first 2 years of life. Further research to understand the contribution specific housing circumstances make to inequalities in LRTI hospitalisations early in life is needed.

